# The Global Genome Biodiversity Network (GGBN) Data Standard specification

**DOI:** 10.1093/database/baw125

**Published:** 2016-10-01

**Authors:** G. Droege, K. Barker, O. Seberg, J. Coddington, E. Benson, W. G. Berendsohn, B. Bunk, C. Butler, E. M. Cawsey, J. Deck, M. Döring, P. Flemons, B. Gemeinholzer, A. Güntsch, T. Hollowell, P. Kelbert, I. Kostadinov, R. Kottmann, R. T. Lawlor, C. Lyal, J. Mackenzie-Dodds, C. Meyer, D. Mulcahy, S. Y. Nussbeck, É. O'Tuama, T. Orrell, G. Petersen, T. Robertson, C. Söhngen, J. Whitacre, J. Wieczorek, P. Yilmaz, H. Zetzsche, Y. Zhang, X. Zhou

**Affiliations:** 1 Botanic Garden and Botanical Museum Berlin-Dahlem, Freie Universität Berlin, Königin-Luise-Str. 6-8, Berlin 14195, Germany; 2 National Museum of Natural History, Smithsonian Institution, Washington, DC 20560, USA; 3 Natural History Museum of Denmark, University of Copenhagen, Sølvgade 83, opg. S, Copenhagen DK-1307, Denmark; 4 Damar Research Scientists, Damar, Drum Road, Cuparmuir, Fife KY15 5RJ, UK; 5 Leibniz Institute DSMZ - German Collection of Microorganisms and Cell Cultures, Inhoffenstr. 7B, Braunschweig 38124, Germany; 6 Australian National Wildlife Collection, CSIRO National Research Collections Australia, Canberra, Australia; 7 Berkeley Natural History Museums, University of California at Berkeley, Berkeley, CA 94720, USA; 8 Global Biodiversity Information Facility Secretariat, Universitetsparken 15, Copenhagen DK-2100, Denmark; 9 Australian Museum, Sydney 2010, NSW, Australia; 10 Systematic Botany, Justus Liebig University, Giessen 35392, Germany; 11 Department of Life Sciences & Chemistry, Jacobs University Bremen gGmbH, Campus Ring 1, Bremen 28759, Germany; 12 Microbial Genomics and Bioinformatics Research Group, Max Planck Institute for Marine Microbiology, Celsiusstrasse 1, Bremen 28359, Germany; 13 ARC-Net Applied Research on Cancer Centre, Department of Pathology and Diagnostics, University of Verona, Verona 37134, Italy; 14 Natural History Museum, Cromwell Road, London SW7 5BD, UK; 15 Department of Medical Informatics and UMG Biobank, University Medical Center Göttingen, Robert-Koch-Str. 40, Göttingen 37075, Germany; 16 Museum of Vertebrate Zoology, University of California at Berkeley, Berkeley, CA 94720, USA; 17 Julius Kuehn-Institute (JKI), Federal Research Centre for Cultivated Plants, Institute for Resistance Research and Stress Tolerance, Erwin-Baur-Str. 27, Quedlinburg 06484, Germany; 18 China National GeneBank, BGI-Shenzhen, Shenzhen, Guangdong 518083, China

## Abstract

Genomic samples of non-model organisms are becoming increasingly important in a broad range of studies from developmental biology, biodiversity analyses, to conservation. Genomic sample definition, description, quality, voucher information and metadata all need to be digitized and disseminated across scientific communities. This information needs to be concise and consistent in today’s ever-increasing bioinformatic era, for complementary data aggregators to easily map databases to one another. In order to facilitate exchange of information on genomic samples and their derived data, the Global Genome Biodiversity Network (GGBN) Data Standard is intended to provide a platform based on a documented agreement to promote the efficient sharing and usage of genomic sample material and associated specimen information in a consistent way. The new data standard presented here build upon existing standards commonly used within the community extending them with the capability to exchange data on tissue, environmental and DNA sample as well as sequences. The GGBN Data Standard will reveal and democratize the hidden contents of biodiversity biobanks, for the convenience of everyone in the wider biobanking community. Technical tools exist for data providers to easily map their databases to the standard.

**Database URL:** http://terms.tdwg.org/wiki/GGBN_Data_Standard

## Introduction

This article provides the background, context, baseline and justification for a data standard developed by the Global Genome Biodiversity Network (GGBN). The standard serves to exchange and share information (data) related to the creation of, maintenance of, and legal provisions connected to physical genomic samples in biodiversity repositories, as well as molecular sequences, data often described as sample metadata. The use of terms in this article is as defined in (1). Additional terms are defined in Table 1. The standard complements other community standards such as Darwin Core (DwC, (2)), Access to Biological Collection Data (ABCD, (3)), and minimum information about any (*x*) sequence (MI*x*S, (4)). The full GGBN Data Standard is available in several notations on the Internet at http://terms.tdwg.org/wiki/GGBN_Data_Standard.

**Table 1.** Explanation of specific terms used in this article

Term	Explanation
Genomic sample	Any biological material preserved to keep its molecular properties (in general excluding human material). Examples include DNA, RNA, tissue and environmental sample (see (1))
Genome quality	High-molecular weight DNA or RNA (see (1))
Material sample	The physical result of a sampling (or subsampling) event. In biological collections, the material sample is typically collected, and either preserved or destructively processed. (see http://terms.tdwg.org/wiki/dwc:MaterialSample)
Environmental sample	A material sample that (i) represents taxonomic biodiversity from across the tree of life (e.g. blood, gut), (ii) represents abiotic substrate or environment (e.g. soil, water, ice core) or (iii) an assemblage of both
Environmental DNA	The physical result of DNA extraction of an environmental sample containing DNA of multiple taxa. Often completely consumed during sequencing
Ancient environmental DNA	The physical result of DNA extraction of an environmental sample older than 100 years (e.g. teeth) containing DNA of multiple taxa. Usually completely processed during sequencing
Tissue sample	A material sample dedicated to a single taxon (e.g. leaf, muscle, leg), often chemically or physically treated to preserve biomolecules from degrading. May contain tissue/DNA of other taxa, e.g. endosymbionts, pathogens, destruents
Genomic DNA sample	The physical result of DNA extraction of a tissue sample containing DNA from a single taxon. Usually not completely consumed during sequencing and deposited in a biodiversity biobank
Ancient genomic DNA sample	The physical result of DNA extraction of a tissue sample older than 100 years (e.g. bones) containing DNA from a single taxon. Usually not completely consumed during sequencing and deposited in a biodiversity biobank

## Background

### Why a network of biodiversity biobanks?

The polymerase chain reaction (5) and Sanger sequencing (6) radically changed modern biology by unlocking the resources stored in natural history museums such as herbaria and zoological collections, as well as culture collections, seed banks, zoos, aquaria and botanical gardens, and encouraging targeted sampling of species and populations in the field. High-throughput sequencing (HTS) methods have been developed and improved (7, 8) and these technologies have enabled the mobilization of even larger parts of these collections with the prospect of future technological developments promising further opportunities. Despite the unprecedented power of new molecular techniques, working with traditionally stored material remains cumbersome. The DNA of specimens in collections is often fragmented due to historical preservation techniques that failed to inhibit endo- and exonuclease activity, or because the DNA has become inaccessible due to preservatives and fixatives that cause extensive post mortem damage, interfering with sequencing (e.g. by cross-linking DNA and proteins in formalin-preserved tissues—see (9)).

To enable new research goals, biodiversity repositories have adapted to accommodate high-quality genomic DNA (i.e. high molecular weight) samples that overcome these barriers. A new approach of measuring genome quality is given in (10). Given millions of known species, and many more millions of unknown species, the effort of assembling synoptic samples of this diversity significantly surpasses the capability of any single institution. Furthermore, because species are disappearing at an unprecedented and steadily increasing rate (11), the need for coordinated sampling efforts, storage and documentation strategies, and data and sample quality management increases. The best way to achieve a synoptic sample of life on earth is to share this task and to collectively make resources available to the wider scientific community. It was with this spirit that the Global Genome Biodiversity Network (GGBN) was created in 2011.

### The GGBN

The GGBN (http://www.ggbn.org) is based on a Memorandum of Cooperation and is an unincorporated, international network of member organizations, which share the aim of making high-quality, well-documented, and vouchered genomic samples of the Earth’s biodiversity discoverable for research. The mission and objective of GGBN is to foster collaborations among biodiversity repositories in order to comply with quality standards, best practices, interoperability and exchange of material in accordance with national and international legislation and conventions, thereby benefiting society through additional research contributing to development and biodiversity conservation. Currently, the network focuses on DNA and tissue banks housed in traditional natural history or culture collections, but membership to the network is open to any biodiversity biobank (e.g. seed banks, crop or animal genetic resource banks, gene banks and other types of biological repositories, as well as representatives of government, academic and other organizations involved in genomic biodiversity). Members are expected to have interests in (i) genomic research and research infrastructure connected to biodiversity and the environment, (ii) interacting with other members and the GGBN Secretariat and (iii) contributing to the achievement of GGBN goals.

Most GGBN founding member institutions are natural history collections and the majority of their research is related to non-human DNA. GGBN’s primary goal is long-term storage and enabling discoverability of tissue and physical DNA (genomic DNA), as well as the associated voucher specimens to allow verification of previous determinations in the future. To understand biodiversity and ecosystems global reference lists of genomic information on all organisms of the Tree of Life are essential. This can only be achieved by combining morphological and molecular approaches. GGBN and its member collections aim at enabling standardized access to vouchered and identified genomic samples as the basis for, e.g. sequence reference lists, including environmental samples. Traditionally, identification of an organism was based only on morphology, often involving special preparations, e.g. genitalia, micro-morphology or CT scans. However, molecular based identification is increasingly important for many taxonomic groups because it offers standardized, automated biodiversity identification. To reach that goal, well-documented reference databases are required to enable automated sequence comparison, e.g. by BLAST (Basic Local Alignment Search Tool, (12)) against Nucleotide Collection (nt) or primary sequence databases operating in the International Nucleotide Sequence Database Collaboration (INSDC, (13); see, however, (14)). This becomes especially important as HTS technologies enable the identification of the contents in environmental samples bypassing pre-sorting and/or pre-cultivation processes. In eDNA studies (environmental DNA) the entire sample is often consumed during DNA extraction or the remaining sample is not stored in public collections, and no morphologically recognizable remains exist to serve as a voucher. The uneven and irregular amplification of DNA during whole genome amplification methods do not alleviate this problem. Indeed quantitatively biased amplification can introduce drastic bias (15). Furthermore, an eDNA sample often contains many, occasionally thousands, of organisms and sequence reads generated from a single sample can be in the order of multi-millions. However, identification queries often fail, primarily due to the lack of reference databases. Most taxa, even in the well-studied areas of Northern Europe, are not represented in reference databases (16).

### The GGBN platform

The GGBN Data Portal [http://data.ggbn.org, (1)] improves the discoverability and use of genomic samples and data by providing standardized access to genome-quality samples and related data from across the Tree of Life. The portal bridges the gap between biodiversity repositories, sequence databases, and research results by linking globally distributed biodiversity databases of genomic samples to vouchered specimens, sequence data, and publications. This infrastructure will: (i) allow a quick assessment of whether adequate samples are available and accessible for a specific project; (ii) identify gaps in our current sampling of the Tree of Life and (iii) guide future, strategic sampling, thus providing necessary tools to save the genetic blueprint of key threatened biodiversity. In addition, the Data Portal enables researchers worldwide to easily request DNA or tissue samples.

Important projects with several thousand genomic samples are already available through GGBN such as Birds 10K Genome (17), birds within Barcode of Life (18), German Barcode of Life [GBOL, (19)] and the Genomic Encyclopedia of Bacteria and Archaea [GEBA, (20)].

Best Practices and Standard Operating Procedures are required to document the processing of genomic samples from the start (e.g. sampling methods in the field) as different communities require varying protocols and knowledge. The GGBN Library (https://library.ggbn.org) enables collaboration between different biodiversity sectors and offers a platform to find and share relevant protocols and methods between communities.

### Biodiversity biobanks

In the 1980s new types of biodiversity repositories, DNA and tissue banks (21–23) emerged. These were developed *ad hoc* across various communities and informed by the OECD’s Biological Resource Centres framework (24) and Best Practice Guidelines (25), and they have become the operational model for the life sciences and biotechnology sector. Today many biodiversity repositories (often as part of natural history collections) store thousands of tissue or DNA samples, but only a tiny fraction of these are registered in a database or linked to an accompanying voucher specimen [see, e.g. (1)], and even fewer are publically available. Often they are stored in different databases not shared among departments even within the same institution. This differs from culture collections, where genomic samples derived from bacterial or cell cultures are commonly well-documented and well-described [e.g. German Collection of Microorganisms and Cell Cultures (DSMZ), Belgium Coordinated Collections of Microorganisms (BCCM)), though the accompanying data are often held in specialized but rarely synchronized databases. Of the 50 current GGBN members, 17 share their data via the GGBN Data Portal, though usually each collection has mobilized only a small fraction of their entire collections. Further collaboration of biodiversity biobank-holding institutions is urgently required to reduce replication of efforts, to maximize access to research resources, and to facilitate responsible and ethical use of collections.

### Collection data sharing—unlocking the hidden treasures

**Figure 1.**
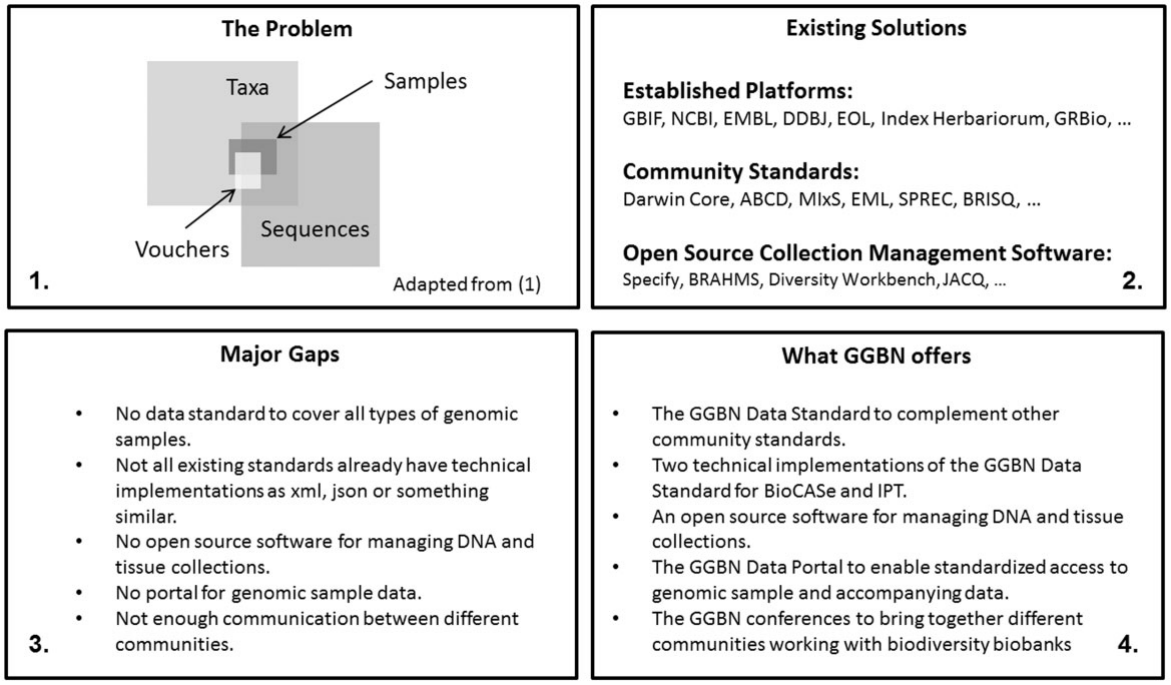
Bridging the gaps. Schematic representation of (1) Low percentage of available sequence data in public repositories with proper information where the voucher and/or sample is deposited. (2) Existing tools and platforms for standardized management and access to biodiversity data. (3) Major gaps identified by GGBN and (4) what GGBN has developed to fill these gaps.

For centuries, biological collections have been an indispensible resource for various biological research activities, as they cover a large part of global biodiversity. Over the past twenty years, data mobilization and digitization efforts have enabled access to many of the billions of specimens accumulated [e.g. Global Biodiversity Information Facility (GBIF, http://www.gbif.org), Integrated Digitized Biocollections (iDigBio, https://www.idigbio.org/) and Atlas of Living Australia (ALA, http://www.ala.org.au)]. To date, digitized records represent only a fraction of the total of specimens. Open access to these has already proven to be vital, allowing researchers worldwide to search for, and digitally reason on, specimens and data. Figure 1 gives an overview about the role of GGBN and proposed solutions to fill major gaps.

Many scientists deposit their specimens in publicly available collections to ensure reproducibility, verification and reference for future research. However, access to data derived from this stored material makes the following implicit assumptions:

- Institutions will be responsible for the biological material that they share. Clear policies are needed on how to handle sensitive data (e.g. indigenous knowledge, endangered species, intellectual property, binding transnational agreements). The GGBN Data Standard can share information at many levels, e.g. not only through public portals, but also via internal networks and inside institutions.
- Information made available to the public will meet robust data standards to assure the highest accuracy and avoid misinterpretation.
- Access and benefit sharing as envisioned in the Nagoya Protocol (26) will require modern levels of data protection that likely to impinge on any biodiversity repository.

### Legal and political considerations with respect to data sharing

The transfer of genomic sample information from initial collection and preservation in the field into collection management systems, along with voucher/strain and transaction/permit data, requires management. Adding and curating identifications made by taxonomic specialists, and linking them to long-term genomic sample storage systems and finally enhancing these with laboratory results is an additional responsibility.

Any data standard for biological material must consider the documentation requirements necessary to comply with legal obligations including those recently set by international bodies. The Convention on Biological Diversity, ratified by 195 countries and the EU, has as one of its three main objectives *fair and equitable sharing of benefits arising from the utilization of genetic resources* (Access and Benefit-Sharing—ABS; https://www.cbd.int/abs/). ABS is the rationale for the Nagoya Protocol (https://www.cbd.int/abs/about/ratified, 12 October 2014), a legally binding agreement between all that have ratified it. Researchers who collect new specimens or genomic samples *in situ* (‘access’) may have to obtain not only the traditional collection permits, but also agree on conditions for use (Prior Informed Consent (PIC) and Mutually Agreed Terms (MAT)), before embarking on a collecting trip. This may *inter alia* include data sharing, capacity building or joint research or, if commercial activities are planned, monetary arrangements. Such agreements persist and may or may not be transferable, and anyone subsequently wanting to carry out research on any of these samples may have to approach the Providing Country to obtain the right to use the material (e.g. a new PIC and MAT). These agreements may limit future activities and uses. Countries ratifying the Nagoya Protocol are responsible for monitoring utilization by researchers and others under their jurisdiction, and for reporting through the ‘ABS Clearing-House’ (https://absch.cbd.int/) on that utilization. While respecting national law, GGBN requires that its members comply with the provisions of the Nagoya Protocol. Thus researchers, collection-holding institutions, and networks should adopt a common Best Practice approach to manage ABS, as has been developed by GGBN. A Code of Conduct; recommendations for implementing the Code of Conduct (the Best Practices), and implementation tools, such as standard Material Transfer Agreements (MTA) and mandatory and recommended data fields in collection databases, are tools which will aid compliance (27). All of these documents are available at the GGBN web site. Adoption of best practices is expected. The GGBN is collaborating with other organizations, such as the Consortium of European Taxonomic facilities (http://www.cetaf.org) to harmonize best practices across overlapping scientific networks. Institutions must agree on specific data and metadata fields in records to ensure that information regarding conditions of access and necessary legal documents are associated with records in the original specimen or genomic sample records; this will both facilitate compliance with those conditions and enable reporting on utilization to be done efficiently. Effective management of the implemented Nagoya Protocol and Access legislation of provider countries by collections will provide transparency and verifiability for the use of genetic material. Collections play a key role here to facilitate and guarantee long-term deposition and availability. This will also have implications beyond natural history collections and be of importance to the entire life science research community.

### A common data standard for a global network of biodiversity biobanks

Standardized material sample information will power a community-driven network to act as a knowledge platform and a stakeholder on a global scale to broker standards related to genomic samples.

In addition to natural history collections, hundreds of repositories deal with cultures, crops, agricultural pests, human parasites, veterinary banks, forensics and ancient samples from humans and domestic animals. Different communities own these collections, but share many of the problems related to material sample handling and documentation. Yet, all approaches to biobanking have one thing in common: they link physical samples to data that describe: (i) the context of collection (its metadata, e.g. place of origin, sample material, loan, permit), (ii) its identification (assignment to relevant biological units in the taxonomic hierarchy) and (iii) its subsequent treatment and analysis (e.g. preparation, preservation, amplification, sequence reads, marker data). INSDC has established a platform for DNA and protein sequence information for non-human and human data [e.g. (28, 29)]. Depositing data at INSDC is a standard requirement for publications today. Despite this requirement, no data standard is available to support standardized exchange of information about available physical genomic samples, such as DNA, tissues or other types of samples for the complete range of repositories mentioned above.

In order to support documentation and enable access to the rapidly growing collections distributed among the network members and to facilitate communication about their content, a globally agreed standard for sharing genomic data is needed.

## Developing the data standard

### Drawing on developments in the larger biobank community

In addition to different biodiversity communities, the GGBN has also drawn on data experts in the larger community of human biobanks using the dbGaP—the database of Genotypes and Phenotypes, with data from genome-wide association studies and medical sequencing (30) and, ENCODE—the Encyclopedia of DNA Elements, with data regarding functional elements in the human genome (31).

In recent years, a sector of human biobanks related to the medical/clinical community has developed two main data standards. The first standard is BRISQ [Biospecimen Reporting for Improved Study Quality, (32)], a 3-tier standard to better understand, interpret, compare and reproduce experimental results, which involve human biospecimens. Since spring 2013, *Nature Genetics* and, since 2014, *Biopreservation and Biobanking* both recommend using BRISQ to describe research biospecimens in publications. The second standard is SPREC [Standard PREanalytical Codes, (33, 34)], a standard to share pre-analytical data (under biobank control) related to collection processing and storage through assigned codes.

### Complementary data exchange standards within the biodiversity community

Adaptations of SPREC to non-human genomic samples have been proposed by Benson *et al.* (35) and Harding *et al.* (36) to provide pre-analytical codes for sample collection and transit, culture initiation, and cryostorage elements. This takes into account a two level SPREC code, where the first describes elements assigned to sample collection, initiation, and processing before storage, and the second comprising elements for cryostorage and recovery, SPREC (A-02).

With the MIxS, the Genomic Standards Consortium (GSC) established a unified standard and single point of entry for describing and sharing standardized information about sequence data from all domains of life. MIxS “minimum information about any (x) sequence” is based on—the “minimum information about a genome sequence” (MIGS) and the “minimum information about a metagenome sequence” (MIMS) (37). MIxS also introduced the “minimum information about a marker gene sequence” (MIMARKS). In addition, the concept of “environmental packages” allows describing the environment from which a sample originates for any sequence.

Within the community of natural history collections two main collection data exchange standards have been developed in the last 15 years: Darwin Core (DwC) and ABCD. Both have been accepted as official TDWG (Taxonomic Database Working Group, Biodiversity Informatics Standard) standards in 2005 and 2007, respectively. Today they are used in, and supported by, biodiversity portals such as GBIF, GGBN, ALA, and BioCASe (Biological Collections Access Service) to share >650 million biodiversity records (occurrences). Since the beginning of 2013, the academic publishing company, *PenSoft*, requires the Darwin Core for all of its journals to describe specimens and observations in submitted publications.

Similar to ABCD and DwC there exists a well-established collection data exchange standard for microorganisms: MCL [Microbiological Common Language, (38)]. This does not include terms for DNA, but it can for the most part be mapped to ABCD and DwC.

### Development and scope of the GGBN data standard

Both DwC and ABCD lack terms for molecular data. Thus in 2007 GGBN, as part of the precursor project DNA Bank Network, began developing a standard for biodiversity DNA biobanks accompanying natural history and culture collections. The result was the DNA extension of ABCD, ABCDDNA (39, 40). Between 2012 and 2015, the GGBN has undertaken major revisions of ABCDDNA and has included other existing standards related to molecular data or tissue data. The outcome is the GGBN Data Standard that incorporates all molecular terms of MIxS, and can also handle SPREC and large parts of BRISQ.

The GGBN Data Standard (http://terms.tdwg.org/wiki/GGBN_Data_Standard) is a set of terms and controlled vocabularies (Table 2) designed to represent sample facts. It does not cover e.g. scientific name, geography or physiological facts. This allows combining the GGBN Data Standard with other complementary standards.

**Table 2.** Vocabularies used within the GGBN Data Standard. basisOfRecord and materialSampleType serve as top level classification for each record

Vocabulary	Description
basisOfRecord/RecordBasis	Darwin Core/ABCD term: The specific nature of the data record. Controlled vocabulary: PreservedSpecimen, FossilSpecimen, LivingSpecimen, HumanObservation, MachineObservation, MaterialSample
materialSampleType	Classification of kind of physical sample in addition to BasisOfRecord/RecordBasis and Preparation Type. Recommended vocabulary: tissue, culture strain, specimen, DNA, RNA, Protein, environmental sample
Material Sample	basic lab facts about a physical DNA or tissue sample; contains terms from MIxS and terms matching some of those in BRISQ Tier 1
Loan	aspects of loan information on specimens, tissue or DNA samples
Permit	legal aspects of sample acquisition, loans and use
Preparation	aspects of specimen or tissue sample preparation or DNA extraction (handled as a preparation); contains terms from SPREC and terms matching some of those in BRISQ Tier 1
Preservation	aspects of sample preservation in a physical collection; contains terms matching some of those in BRISQ Tier 1
Amplification	aspects of amplification, sequencing and genetic accession numbers; contains terms from MIxS
DNA Cloning	aspects of DNA cloning and NGS libraries; contains terms from MIxS
Gel Image	gel image facts
Single Read	aspects of a single read, including chromatograms and primers

Technically, the standard is documented in a Semantic Mediawiki to allow simultaneous usage within Semantic Web technologies (Linked Open Data) such as RDF (Resource Description Framework) or SKOS (Simple Knowledge Organization System) as well as a human readable documentation. Potentially, the standard can be used not only for non-human genomic samples but also human samples.

Table 2 Vocabularies used within the GGBN Data Standard. basisOfRecord and materialSampleType serve as top level classification for each record.

### Implementation

MIxS, BRISQ and SPREC can be perceived as sets of vocabularies for a certain topic and community. A JSON-based toolkit, including a JSON Schema and validation services, for MIxS (41) is currently under development. SPRECware (42) is a software provided by ISBER (International Society for Biological and Environmental Repositories) to create the SPREC code. The BRISQ Report tool is a software provided by Canadian Tissue Repository Network to provide the relevant biospecimen related data as a structured report, and to promote its inclusion as supplementary material in publications (43). Today DwC is used mainly either as an XML Schema with queries and resulting XML documents transmitted and retrieved via a protocol such as the TAPIR protocol (TDWG Access Protocol for Information Retrieval, http://tdwg.github.io/tapir/docs/tdwg_tapir_specification_2010-05-05.html) or as field delimited files in a zipped DwC Archive with the (IPT) Integrated Publishing Toolkit (44). ABCD is used as an XML Schema with the BioCASe protocol and Provider Software (40). In addition, BioCASe Provider Software supports Darwin Core Archive and can handle any XML schema (e.g. MCL). GGBN has developed two implementations for ABCD (ABCDGGBN and ABCDGGBN-Enviro) and one for Darwin Core. The GGBN Data Standard is fully supported by BioCASe version 3.5.3 (http://www.biocase.org), and IPT version 2.2 (http://www.gbif.org/ipt).

**Figure 2.**
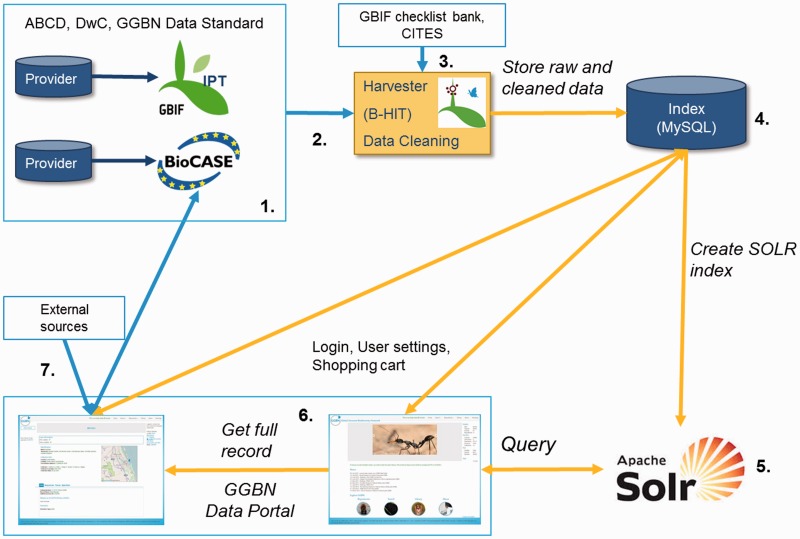
Implementation of the GGBN Data Standard within the GGBN Data Portal. (1) Data are provided by our members by using the GGBN Data Standard with BioCASe or IPT. (2) Harvesting of records with B-HIT occur in compliance with mandatory and highly recommended terms defined by GGBN. (3) Scientific Names are checked against the GBIF checklist bank (http://www.gbif.org/dataset/search?type=CHECKLIST) and CITES (Convention on International Trade in Endangered Species of Wild Fauna and Flora, https://www.cites.org/). In addition to the (4) MySQL database of B-HIT a (5) SOLR instance (http://lucene.apache.org/solr/) is used to speed up queries. Finally, (6) the full record is displayed in the portal with all GGBN Data Standard terms provided by the respective repository as well as associated voucher specimen information, multimedia data, and related information from (7) external sources such as GBIF and INSDC.

The GGBN Data Portal infrastructure is built on B-HIT [Berlin Harvesting and Indexing Toolkit, (45)] to harvest both BioCASe and IPT records in compliance with the GGBN Data Standard (Figure 2).

### Conclusion and outlook

Mobilizing currently scattered DNA and tissue samples by providing access to their data through a common platform will boost research, democratize their use, and make the Convention on Biological Diversity ABS a reality. Molecular research results in an exponentially increasing number of sequences and hundreds and hundreds of publications every year. Knowledge of biodiversity biobank content is urgently needed to enable concerted efforts and strategies in collecting and sampling new material. The GGBN Data Portal provides an infrastructure for making genomic samples discoverable and accessible, as well as enabling gap analysis. However, this can only be achieved by a shared data standard and standardized practices and policies.

The strong emphasis on natural history and culture collections in biodiversity biobanking is a logical extension of these institutions’ traditional focus on all aspects of taxonomy/systematics. The advent of molecular techniques has added new aspects to classical disciplines including species identification (e.g. DNA barcoding) and boosted phylogeny (e.g. sequence variation). HTS is mobilizing a larger and larger part of the traditional collections and had led to a new research field, museomics, and transformed research in many fields, e.g. genomics, transcriptomics, conservation genomics, phenomics, phylogenomics, etc. Still, awareness of, and access, to high-quality DNA makes life easier for any of these disciplines. Wide availability of data will also encourage new initiatives to strategically increase coverage of the Tree of Life among collections.

The GGBN Data Standard complements existing community standards and therefore can serve as an outstanding solution to a major problem: the lack of discoverability and accessibility of genomic samples, and associated voucher specimen information for biodiversity research. Lack of this knowledge significantly limits the types and number of research questions that can be asked and leads to unnecessary sampling of taxa/regions already represented in existing collections. Discoverability and access to genomic samples remains a bottleneck for research that the GGBN Data Standard alleviates. Without the Data Standard, there would be no common vocabulary for sharing data. Data shared using the GGBN Data Standard and associated tools are among the important products meeting the need for properly documented quality data about DNA and tissue samples. In addition to sharing available genetic materials, the effort to coordinate these data flows can become a significant and practical impetus for better communication among institutions and different collection communities, to support compliance with the Nagoya Protocol.

Today 20 GGBN member institutions have already adapted their databases to comply with the GGBN Data Standard. This includes all 17 core members, as well as Australian National Wildlife Collection, South Australian Museum Australian Biological Tissue Collection and Museum Victoria. Not all parts of the standard are relevant for all members, but some are mandatory, e.g. the Permit vocabulary. GGBN provide examples and best practice guidelines through its library (e.g. https://library.ggbn.org/share/s/ky6kLqo-QW2cWKIZVvR7GA) and wiki (e.g. http://wiki.ggbn.org/ggbn/Mandatory_and_recommended_fields_for_sharing_data_with_GGBN).

The GGBN Data Portal already aggregates data from many sources to enrich its data, e.g. web services from GBIF, NCBI (National Center for Biotechnology Information, http://www.ncbi.nlm.nih.gov/), EOL (Encyclopedia of Life, http://www.eol.org), etc. Together with its partners GGBN will continue to work on improving crosslinks between existing platforms to fulfill the needs of communities working with molecular data. This includes taxonomic backbones (e.g. GBIF checklist bank), the use of stable identifiers for every object, as well as transparent information on terms of use and origin of data and samples across all platforms. Only if data from all biological sample types across the Tree of Life are connected and available we will be able to meet the challenges of understanding biodiversity.

All communities involved in biobanking benefit tremendously through mutual interaction (46). GGBN will continue to bridge the gap to other communities to improve knowledge and data exchange or cross-references between different platforms such as GGBN and INSDC (e.g. the BioSample project, sequence submission automation). GGBN has submitted the GGBN Data Standard to the GSC to be endorsed as an official GSC project. In addition, the Data Standard has been submitted to the TDWG committee to be ratified as an official data standard within the natural history collections community.

We envision GGBN growing rapidly into a self-sustaining entity, as institutions and scientists across the globe realize the importance of its ultimate goals—to share a blueprint for developing an intelligently sampled cross section of the Tree of Life for current research and for the benefit of generations of researchers to come. The GGBN Data Standard is one of the core GGBN tools to reach this goal.
